# Hospice care self-efficacy among clinical medical staff working in the coronavirus disease 2019 (COVID-19) isolation wards of designated hospitals: a cross–sectional study

**DOI:** 10.1186/s12904-020-00692-0

**Published:** 2020-12-10

**Authors:** Ze-hong Zheng, Zhong-chen Luo, You Zhang, Wallace Chi Ho Chan, Jian-qiong Li, Jin Pang, Yu-ling Jia, Jiao Tang

**Affiliations:** 1 Guizhou Minzu University, Huaxi, Guiyang, China; 2 School of Nursing, Guizhou Medical University, Guiyang, China; 3 School of Foreign Languages, Chongqing Medical University, Chongqing, China; 4 Department of Social Work, The Chinese University of Hong Kong, Shatin, Hong Kong, SAR China; 5 School of Nursing, Chongqing Three Gorges Medical College, Tianxing Road, Chongqing, China; 6 Nursing Department, Guizhou Provincial People’s Hospital, Guiyang, China; 7 School of Nursing, Chongqing Medical University, 1#, Medical College Road, Chongqing, 400016 China

**Keywords:** Coronavirus disease 2019 (COVID-19), Hospice care self-efficacy, Medical staff, Self-competence in death work

## Abstract

**Background:**

The COVID-19 pandemic has caused more than 462,417 deaths worldwide. A large number of patients with severe COVID-19 face death in hospital. Hospice care is truly a philosophy of care that delivers patient-centred care to the terminally ill and their families. Hospice care could provide many benefits for patients, families, and for hospice caregivers. The aim of this study is to investigate hospice care self-efficacy and identify its predictors among Chinese clinical medical staff in COVID-19 isolation wards of designated hospitals.

**Methods:**

A cross-sectional design was used. The Hospice Care Self-Efficacy, Self-Competence in Death Work Scale, Positive Aspects of Caregiving, and Simplified Coping Style Questionnaires were administered between February and April 2020. A total of 281 eligible medical staff responded to the questionnaires, with a response rate of ≥78.9%.

**Results:**

The mean score of hospice care self-efficacy was 47.04 (SD = 7.72). Self-efficacy was predicted by self-competence in death work (B = 0.433, *P* < 0.001), positive aspects of caregiving (B = 0.149, *P* = 0.027), positive coping (B = 0.219, *P* < 0.001), giving hospice care to dying or dead patients before fighting against COVID-19 (B = -1.487, *P* = 0.023), occupational exposure while fighting against COVID-19 (B = -5.244, *P* = 0.004), holding respect for life and professional sentiment as motivation in fighting against COVID-19 (B = 2.372, *P* = 0.031), and grade of hospital employment (B = -1.426, *P* = 0.024). The variables co-explained 58.7% variation of hospice care self-efficacy.

**Conclusion:**

Clinical nurses and physicians fighting COVID-19 reported a moderate level of hospice care self-efficacy during this pandemic. Exploring the traditional Chinese philosophy of life to learn from its strengths and make up for its weaknesses and applying it to hospice care may provide a new framework for facing death and dying during the COVID-19 pandemic. Continuous hospice care education to improve self-competence in death work, taking effective measures to mobilize positive psychological resources, and providing safer practice environments to avoid occupational exposure are also essential for the improvement of the hospice care self-efficacy of clinical nurses and physicians. These measures help caregivers deal effectively with death and dying while fighting against the COVID-19 pandemic.

## Background

Coronavirus disease 2019 (COVID-19), the third known zoonotic coronavirus disease after severe acute respiratory syndrome (SARS) and the Middle East respiratory syndrome (MERS), is an acute, infectious pneumonia caused by a novel coronavirus [1]. Because of its high transmissibility, strong infectivity, high mortality rate (1–15%) and absence of clinically approved antiviral drug or vaccine, it has become a pandemic and has been seriously endangering human health and life [2–4]. As of 15:00 on September 25, 2020, the World Health Organization (WHO) reported that there were 7,512,285 confirmed cases of COVID-19 and 987,415 deaths, and that number was increasing [5]. A large number of patients with severe pneumonia died in COVID-19 designated hospital or inevitably faced death during the pandemic. Symptoms of patients with severe COVID-19 can escalate rapidly [6, 7], and patients often suffer from anxiety, depression, and insomnia. These symptoms positively correlated with fatigue, dyspnoea, myalgia, and sore throat [8]. In response, a series of emergency medical and psychology plans as well as strategies used to manage deterioration and potential deaths were became necessary [9].

Hospice care generally falls into the category of palliative care [10]. It is a philosophy and system of care for terminally ill patients that allows them to accept death in an affirmative way, and provides palliative care and emotional support for dying patients and their families [11]. Hospice care aims to improve the quality of life rather than its length, and to prepare patients and their families for the end of life [12] by meeting the needs of terminally ill patients through expert symptom management, facilitation of caregiver support, and even provision of home-based care [13]. Hospice care is truly a philosophy of care that embodies the concept of patient-centred care [12, 14]. Evidence showed that hospice care was not only beneficial to terminally ill patients and families (e.g., emotional support, companionship, and practical assistance,), but also to hospice caregivers (e.g., being able to make a difference in the lives of others, personal growth, and greater appreciation of what is really important in life) [15–17].

Hospice care is most often provided at home; however, it can also be provided in an inpatient setting, including hospital, nursing home, or stand-alone hospice facilities. Hospice care requires a multidisciplinary team-based approach to care and relies on families, friends, and other loved ones as well as volunteers to assist in quality care [12]. However, because of the need to control nosocomial infections and make the best use of limited personal protective equipment (e.g., mask, goggles, medical protective clothing, etc.), patients’ family members and other medical personnel who were not responsible for this kind of infectious disease have less chance to intimately contact patients with confirmed COVID-19. To a large extent, instead of a multi-disciplinary team, the clinical nurses and physicians who are involved in fighting against COVID-19 become the main providers of hospice care for dying COVID-19 patients.

Nurses and physicians involved in fighting against pandemics such as the COVID-19 suffer from high physical and mental workloads, stress, and risk of infection [18–20], all of which affect their comfort and health [21, 22]. Researches also reported that nurses might adopt negative attitudes or actions, including avoiding confirmed or suspected cases, when they were involved in the management of patients who are infected or even who died of infectious diseases [23, 24].

Self-efficacy refers to the personal judgment of how well an individual can execute required courses of action to deal with prospective situations, and the hospice care self-efficacy is targeted at hospice care and addressed the health workers’ confidence regarding the provision of mental and spiritual care for the terminally ill and their family members [25]. Studies found that high level of hospice care self-efficacy helps hospice care givers avoid negative emotions (e.g., escape, fear) [26, 27], and actively assume their professional responsibilities [28]. Queries regarding the attitudes and self-efficacy of clinical nurses and physicians involved in fighting against COVID-19 during the implementation of hospice care for patients with dying COVID-19 was the focus aim of this study.

The aim of this study was to investigate hospice care self-efficacy and to identify its predictors among Chinese clinical medical staff in the COVID-19 isolation wards of designated hospitals. The findings may provide clinical managers in China and in other countries with experience on psychological strategies regarding the fight of medical staff against COVID-19 so as to develop more effective strategies to cope with COVID-19 deaths and dying patients.

## Methods

### Study design and setting

This was a cross-sectional questionnaire survey. The data were collected from a large number of clinical medical staff fighting against COVID-19 pandemic between February and April 2020 in China. These medical staff were invited to take part in this study if they: (1) had nurse or physician qualifications granted by the National Health Commission (NHS); (2) had assisted and worked in the COVID-19 isolation ward of COVID-19 designated hospitals, both in Hubei province and in other local provinces. We excluded medical personnel who were not involved in the treatment and care of COVID-19 patients in the isolation wards of designated hospitals, including logistics personnel and medical technicians. Ethical approval was obtained from the Medical Ethics Committee of Guizhou Medical University in Guiyang, China. The research conformed to the provisions of the Declaration of Helsinki in 1995 (as revised in Edinburgh in 2000).

### Data collection

The data were collected by combining a convenience sampling method and a snowballing sampling method. We collected all participant information using the Questionnaire Star, a professional online survey platform developed by Changsha Ranxing Information Technology Co. (China). This survey platform has 33.75 million users and has previously been used to collect 2.334 billion responses to questionnaires in China. The data collection process was as follows:

#### E-questionnaire setting

We edited our in-house e-questionnaire using the platform and generated a link that would provide access to our e-questionnaire via WeChat, an instant mobile messaging software with the largest user groups in China. The questionnaire stated the purpose and methods of our study. The risk of participating in the survey was described to acquire informed consent on the first page, and each participant could decide of their own free will whether to join our survey. They also had the right to quit the survey at any time without any further consequences. If the potential participants were not willing to join or would like to quit the survey, they could directly exit the questionnaire link to drop out. Each participant would also have to answer two questions (‘Are you a clinical nurse or physician with qualification granted by the National Health Commission (NHS)?’, and ‘Have you worked in the COVID-19 isolation ward of designated hospitals?’) at the top of the questionnaire to ensure that the inclusion and exclusion criteria of our study would be met.

#### E-questionnaire distribution

Medical staff who assisted and worked at COVID-19 designated hospitals in Hubei province or their own provinces would be invited to participate in the study by sending the e-questionnaire link to their official WeChat groups. In this step, we invited 23 medical staff (four medical staff who came from Guizhou Province, three from Chongqing municipality, and four from Shanxi Province, Sichuan Province, Inner Mongolia Autonomous Region and Guangzhou city, respectively) who met our selection criteria. These participants were encouraged to send our e-questionnaire link to acquaintances who met these criteria. To increase the number of participants, all participants were given the chance to draw a random digital red packet on WeChat as the reward, and the distributors would receive an additional bonus. Based on these sampling methods, our link was disseminated quickly via a wide network and participants simply needed to click the link and follow the online prompts to complete the questionnaire.

The sample size was determined through power analysis and calculated using the G*Power program [29, 30]. When considering an effect size of 0.15 and 26 related factors [25, 28, 31, 32], significance level (*p*) of 0.05, 95% power, at least 241 participants were required.

**Fig. 1:**
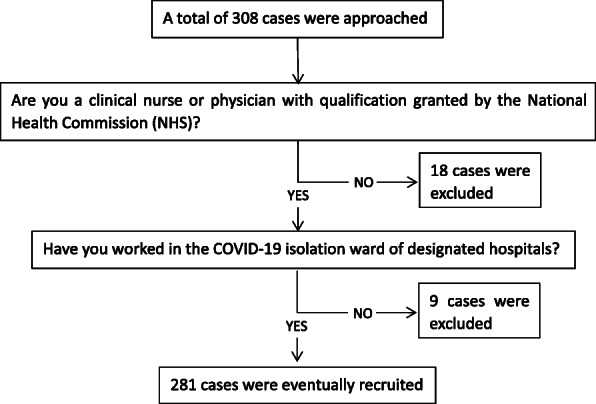
Data collection process diagram

A total of 360 participants joined our study. All submitted the questionnaire; however, 18 were excluded because they were not a nurse or physician (e.g., medical technicians, managers and logisticians), and 61 were not working in the infectious disease isolation ward of COVID-19 designated hospitals (working in departments such as fever outpatient and wards not for COVID-19 patients) (Fig. 1). As shown in Questionnaire Star, there were 435 clicks on our questionnaire link, and we received from 360 respondents. Among them, 281 met the inclusion and exclusion criteria. We could only count the number of times the questionnaire was visited (including the number of visits in which the questionnaire was not ultimately submitted), instead of the exact numbers of visits to their internet protocol address. This means that if a respondent visited our questionnaire more than once, the platform automatically recorded their actual visit number although the visitor represented only one subject. Therefore, our response rate may be equal to or greater than 78.9% (Response rate ≥ 281/(435–[360–281]).

### Measures

#### Hospice care self-efficacy scale

The Hospice Care Self-Efficacy Scale was one of dimensions of Death Coping Self-Efficacy Scale. It was adapted from the Hospice-Related Death Coping Self-Efficacy Scale presented by Robbins [33]. The Death Coping Self-Efficacy Scale included 29 questions and the Hospice Care Self-Efficacy Scale dimensions accounted for 12 questions [25]. Each question item was rated using a 5-point Likert scale. The total scores of the Hospice Care Self-Efficacy Scale ranged from 12 to 60 points. Higher scores indicated a higher level of hospice care competency. The content validity test results of the whole Death Coping Self-Efficacy Scale in the Chinese version indicated that the content validity indices (CVIs) of its three dimensions ranged from 0.40 to 1.00 with an average of 0.87, and the Cronbach’s α of the official Chinese version was 0.88. Nevertheless, it was not a clear independent report of the CVIs or Cronbach’s α of the Hospice Care Self-Efficacy Scale dimensions [25]. In the present study, the Cronbach’s α of the scale was 0.95.

#### Self-competence in death work scale (SC-DWS)

Self-competence in death work was measured using the 16-item SC-DWS, developed and validated in Hong Kong [34]. The authors recommended using the whole scale to report the overall score of self-competence in death work. Participants were asked to rate their responses according to the extent to which the items were compatible with their current situation, on a scale of 1 point (completely incompatible) to 5 points (completely compatible). The rating of each item was summed to form a total score. A higher score represented a higher level of self-competence in death work. The whole scale showed good internal consistency, with Cronbach’s α 0.88 [35, 36].

#### Positive aspects of caregiving (PAC)

PAC was developed by Tarlow in 2004 to measure the positive feelings of caregivers of patients with Alzheimer’s disease [37]. Since then, the scale has been translated into several languages and has been widely used to determine the positive feelings of caregivers of cancer patients, chronic patients, community nurses, and others [38–40]. It includes nine items that were made up of two dimensions. The first five items cover the dimension of self-affirmation and the last four items cover the dimension of life outlook. Five-point Likert ratings were used, with a scale ranging from 1 point (strongly disagree) to 5 points (strongly agree). Higher scores indicated higher positive feelings experienced by the caregiver. Zhang et al. translated the scale into a Chinese version [41]. The whole scale Chinese version, including subscales, showed good internal consistency, with Cronbach’s αs of 0.90, 0.89, and 0.83, respectively, and the content validity test indicated that the CVIs ranged from 0.80 to 1.00 with an average of 0.95.

#### Simplified coping style questionnaire (SCSQ)

SCSQ were adapted by Xie [42] based on the Ways of Coping Questionnaire (WCQ) [43]. It was measured using the 20-item instrument and was divided into a positive coping (12 items) dimension and a negative coping (eight items) dimension, on a 4–point Likert scale ranging from 0 point to 3 points. The SCSQ had adequate content validity, internal consistency, and test–retest reliability in the Chinese version [42]. The Cronbach’s α of the positive coping and negative coping dimensions were 0.89 and 0.78, respectively.

Demographic characteristics were collected using a demographic data sheet. The items ‘work motivation in fighting against COVID-19’ were assessed using multiple-answer questions with eight selections.

#### Statistical analyses

Survey data were exported from Questionnaire Star into the Statistical Package for Social Sciences (SPSS) package (v20.0, IBM, USA), which was used for all data analysis. Descriptive statistics were used to express the sample characteristics and study variables. Differences in the hospice care self-efficacy of participants with different demographics, work motivations in fighting against COVID-19, self-competences in death work, coping strategies and positive aspects of caregiving were assessed using t-tests, or one–way analysis of variance (ANOVA), or the correlation test (continuous variables in this study showing a normal distribution). Variables identified as being significant (*P* < 0.05) in these initial tests were then entered into a multiple linear regression models to determine predictors for the levels of hospice care self-efficacy of medical staff fighting against COVID-19.

## Results

### Socio-demographic characteristics

Table 1 displays the demographic information of our participants. The mean age was 32.96 ± 5.96 years. More than 70.0% participants were females and were married; 63.0% of participants came from level 3 hospitals; however, only 27.0% had ever cared for or treated patients with secondary protection or above prior to this public health emergency. More than half worked in or assisted at COVID-19 designated hospitals in Hubei province, 33.1% in intensive care units for patients with COVID-19. The length of exposure to patients COVID-19 ranged from 1 to 90 days with an average of 33.1 ± 16.27 days.

**Table 1:** Characteristics of the participants (*n* = 281)

Characteristic	Mean ± SD	f	%
**Age (year)**	32.96 ± 5.96		
**Gender**			
Male		70	24.9
Female		211	75.1
**Marital status**			
Unmarried		67	23.8
Married		206	73.3
Divorced or widowed		8	2.8
**Profession**			
Physician		54	19.2
Nurse		227	80.9
**Educational attainment**			
Technical secondary school or Junior College		43	15.3
Undergraduate		208	74.0
Postgraduate or above		30	10.7
**Professional titles**			
Primary		162	57.7
Intermediate		99	35.2
Senior		20	7.1
**Length of work (year)**	10.01 ± 6.26		
**Grade of employing hospital**			
Level 3 hospitals		177	63.0
Level 2 hospitals		104	37.0
**Department of employing hospital**			
Intensive care unit		56	19.9
Others		225	80.1
**Had ever cared or treated any patients with secondary protection or above before fighting against COVID-19**			
Yes		205	73.0
No		76	27.0
**The location of COVID-19 designated hospital**			
Hubei Province		157	55.9
Others Province		124	44.1
**Post in COVID-19 designated hospitals**			
Intensive care unit for patients with COVID-19		93	33.1
Other departments of COVID-19		188	66.9
**Work motivation in fighting against COVID-19 (Multiple response)**			
Respect for life and professional sentiment		257	91.5
Support from leaders or colleagues		169	60.1
Support from family		197	70.1
Expectations for the future		118	42.0
Have confidence in fighting against COVID-19		224	79.7
Government’s policy support		189	67.3
Preferential treatment offered by the employing organization		60	21.4
Others^a^		4	1.4
**Had ever given hospice care for dying or dead patients before fighting against COVID-19?**			
Yes		192	68.3
No		89	31.7
**Have given hospice care for dying or dead patients while fighting against COVID-19?**			
Yes		106	37.7
No		175	62.3
**Had any occupational exposure working in COVID-19 designated hospitals?**			
Yes		8	2.8
No		273	97.2
**Duration of exposure to patients with COVID-19 (day)**	33.11 ± 16.27		
**Working hours per day in COVID-19 designated hospitals**	7.17 ± 3.37		

^a^including ‘I believe motherland is my powerful support so not afraid to get infected’, ‘The Wuhan government provides adequate living security’, ‘Personal Accountability’ and ‘I must stick to it out, it’s not good to quit halfway’

### Self-competence in death work, coping strategy, positive aspects of caregiving and hospice care self-efficacy

Table 2 displays the levels of self-competence in death work (mean = 59.85, SD = 9.63) and positive aspects of caregiving (mean = 38.23, SD = 5.91). Coping strategies (positive and negative coping strategies were mean 4.43 (SD = 6.52) and 10.79 (SD = 5.38) respectively, and hospice care self-efficacy of the participants was mean 47.04 (SD = 7.72).

**Table 2:** Self-competence in death work, positive aspects of caregiving, coping strategies, and hospice care self-efficacy of the participants (*n* = 281)

	Range of total scores	Minimum of actual score	Maximum of actual score	Mean scores (Mean ± SD)
**Self–competence in death work**	16.00–80.00	16.00	80.00	59.85 ± 9.63
Existential coping	10.00–50.00	12.00	60.00	44.77 ± 7.32
Emotional coping	4.00–20.00	4.00	20.00	15.08 ± 2.86
**Positive aspects of caregiving**	9.00–45.00	12.00	45.00	38.23 ± 5.91
**Coping strategies**				
Positive coping	0.00–48.00	0.00	36.00	24.43 ± 6.52
Negative coping	0.00–32.00	0.00	24.00	10.79 ± 5.38
**Hospice care self-efficacy**	5.00–60.00	12.00	60.00	47.04 ± 7.72

### Predicting the level of hospice care self-efficacy among medical staff fighting against COVID-19

Univariate analysis identified a range of factors that were significantly associated with the hospice care self-efficacy of the participants: grade of employing hospital (t = 3.206, *P* = 0.002), professional titles (F = 6.061, *P* = 0.003), self-competence in death work (*r* = 0.701, *P* < 0.001), history of providing hospice care for dying or dead patients before fighting against COVID-19 (t = 2.404, *P* = 0.017), occupational exposure while fighting against COVID-19 (t = 2.404, *P* = 0.017), positive aspects of caregiving (*r* = 0.505, *P* < 0.001), positive coping (*r* = 0.516, *P* < 0.001), negative coping (*r* = 0.208, *P* < 0.001), holding respect for life and professional sentiment (t = − 4.180, *P* < 0.001) and expectations for the futures (t = − 2.342, *P* = 0.020) (Table 3).

**Table 3:** Differences in Hospice care self-efficacy among various demographic sub-groups (*n* = 281)

Characteristic	Hospice care competency		
	Mean ± SD	t/F/r	***P***
**Age (year)**		0.092	0.124
**Gender**		1.817	0.070
Male	48.49 ± 8.65		
Female	46.46 ± 7.35		
**Marital status**		0.506	0.603
Unmarried	46.24 ± 6.95		
Married	47.26 ± 8.03		
Divorced or widowed	48.00 ± 5.73		
**Profession**		−1.00	0.920
Physician	46.94 ± 9.08		
Nurse	47.06 ± 7.38		
**Educational attainment**		2.304	0.102
Technical secondary school or Junior College	47.53 ± 6.95		
Undergraduate	46.55 ± 8.13		
Postgraduate or above	49.70 ± 4.92		
**Professional titles**		**6.061**	0.003*
Primary	45.70 ± 7.81		
Intermediate	48.70 ± 7.53		
Senior	49.65 ± 5.61		
**Length of work (year)**		0.102	0.088
**Grade of employing hospital**		**3.206**	0.002*
Level 3 hospitals	48.15 ± 7.02		
Level 2 hospitals	45.14 ± 8.49		
**The department of employing hospital**		0.537	0.592
Intensive care unit	47.54 ± 9.19		
Others	46.92 ± 7.32		
**Had ever cared or treated any patients with secondary protection or above before fighting against COVID-19**		1.851	0.065
Yes	47.56 ± 8.18		
No	45.65 ± 6.16		
**Have given hospice care for dying or dead patients before fighting against COVID-19**		**2.404**	0.017*
Yes	47.79 ± 6.92		
No	45.43 ± 9.04		
**Had given hospice care for dying or dead patients while fighting against COVID-19**		1.677	0.095
Yes	48.03 ± 7.10		
No	46.44 ± 8.35		
**Had occupational exposure while fighting against COVID-19**		**2.279**	0.023*
Yes	53.13 ± 5.74		
No	46.86 ± 7.71		
**Duration of exposure to patients with COVID-19 (day)**		−0.015	0.804
**Working hours per day in COVID-19 Designated Hospitals**		−0.077	0.199
**Self-Competence in Death Work**		**0.701**	< 0.001*
**Positive coping**		**0.516**	< 0.001*
**Negative coping**		**0.208**	< 0.001*
**Positive Aspects of Caregiving**		**0.505**	< 0.001*
**Work motivation in fighting against COVID-19(Multiple Response)**			
Respect for life and professional sentiment	40.92 ± 8.46	−4.180	< 0.001*
Support from leaders or colleagues	47.54 ± 7.97	−1.350	0.178
Support from family	47.05 ± 8.10	−0.022	0.983
Expectations for the future	48.30 ± 6.68	**−2.342**	0.020*
Have confidence in fighting against COVID-19	47.33 ± 7.71	− 1.235	0.218
Government’s policy support	46.66 ± 7.45	1.176	0.240
Preferential treatment offered by the employing organization	47.33 ± 8.27	−0.332	0.740
Others^a^	50.00 ± 6.58	−0.772	0.441

*Statistically significant in t-test, ANOVA, or correlation test, *P* < 0.05

^a^Others including ‘I believe motherland is my powerful support so not afraid to get infected’, ‘The Wuhan government provides adequate living security’, ‘Personal accountability’ and ‘I must stick to it out, it’s not good to quit halfway’

A best-fit multiple linear regression models identified several significant predictors of the level of hospice care self-efficacy of participants: grade of employing hospital (B = − 1.426, *P* = 0.024), self-competence in death work (B = 0.433, *P* < 0.001), history of having given hospice care for dying or dead patients before fighting against COVID-19 (B = − 1.487, *P* = 0.023), occupational exposure while fighting against COVID-19 (B = − 5.244, *P* = 0.004), positive aspects of caregiving (B = 0.149, *P* = 0.027), positive coping (B = 0.219, *P* < 0.001), and holding respect for life and professional sentiment (B = 2.372, *P* = 0.031). The variables co-explained 58.7% of the variation of hospice care self-efficacy (Table 4).

**Table 4:** Predictors of hospice care self-efficacy (*n* = 281)

Variable	B	SE -B	β	t	***p***	95% CI		VIF
						Low	Up	
Constant	22.139	4.417	/	5.012	< 0.001	13.444	30.835	/
Self-competence in death work	0.433	0.037	0.540	11.826	< 0.001	0.361	0.505	1.414
Positive coping	0.219	0.059	0.185	3.696	< 0.001	0.102	0.336	1.702
Had occupational exposure during fighting against COVID-19	−5.244	1.812	−0.113	−2.894	0.004	−8.810	−1.677	1.037
Grade of hospital	−1.426	0.628	−0.089	−2.270	0.024	−2.663	−0.189	1.051
Respect for life and professional sentiment was the work motivation in fighting against COVID-19	2.372	1.096	0.086	2.165	0.031	0.215	4.529	1.071
Have ever given hospice care for dying or dead patients before fighting against COVID-19	−1.487	0.649	−0.090	−2.292	0.023	−2.764	−0.210	1.040
Positive aspects of caregiving	0.149	0.067	0.114	2.221	0.027	0.017	0.281	1.792

*Abbreviations*: *B* unstandardized coefficient beta, *SE -B* standard error of B, *β* standardized coefficient beta, *CI* Confidence Interval, *VIF* variance inflation factor

## Discussion

To our best knowledge, this was the first study to investigate hospice care self-efficacy among caregivers treating fatal infectious diseases in mainland China. Hospice care has been reported as being an effective measure to improve the life quality of dying patients and to help their families cope with bereavement [12]. Medical staff employing effective hospice care might avoid the adverse effects of sleep disorders, irritability, interpersonal problems, and other issues [44]. For these reasons, it is essential to investigate hospice care self-efficacy and to identify its predictors among clinical medical staff involved in fighting against the COVID-19 pandemic.

In this study, clinical nurses and physicians reported moderate levels of hospice care self-efficacy while offering care to dying COVID-19 patients. The COVID-19 pandemic led to the emergence of a large number of confirmed patients in a short period of time [5], which further led to shortages of medical supplies (e.g., medical protective equipment, etc.) and health professionals. Communication was difficult between medical staff and patients because the latter needed to wear personal protective equipment and in some cases patients were delirious or had hearing or sight impairments [9]. More importantly, nurses and physicians who were involved in fighting against COVID-19 pandemic suffered from heavy workloads and stress [18, 19], which might further restrict the time and energy that they could have spent implementing hospice care.

These findings may be related to the Chinese traditional philosophy of life that includes the ethical thoughts in traditional Chinese culture, including Confucianism (e.g., paying attention to the present world and pursuing living forever), Taoism (e.g., believing that life and death are unified and life is immortal), and Buddhism (e.g., deeming that individuals are reincarnated without extinction) [45]. The traditional Chinese philosophy of life respects the natural law of death and values life; however, it places taboos on death and attaches great importance to the continuation of life. To some extent, Chinese traditional philosophy of life is dissonant with the concept of hospice care, and this could affect the development and implementation of hospice care in China. Therefore, exploring the traditional Chinese philosophy of life to learn from its strengths and compensate for its weaknesses and examining the psychology of hospice care providers may be conducive to providing new directions for the integration of dilemmas faced by hospice care providers in the context of infectious diseases characterized by high infectivity and mortality.

We found that nurses and physicians with higher self-competence in death work had better hospice care self-efficacy. Self-competence in death work refers to ‘the competence required to cope with the emotional and existential challenges to self in working with death or matters related to death’ [46]. Assessing self-competence in death work among hospice care professionals may help to better reflect their needs in facing death [34]; successfully development of self-competence in death work may improve attitudes and self-efficacy, resulting in better job performance of hospice care, especially for those who had early experiences with patient death [44, 47]. A systematic review and qualitative meta-synthesis found that continuous education regarding how to face and accept death would promote hospice care professional growth among nurses [32]. Hospice care education, especially scenario simulations to place medical staff into simulated bereavement or death situations and to allow them to become aware of their personal needs in facing death, were used urgently to improve medical staff self-competence and self-efficacy in the death work associated with treating emerging infectious diseases [32, 35].

Nurses and physicians who had acquired hospice care experience by giving hospice care for dying or dead patients in a hospital or hospice prior to the COVID-19 pandemic also had better hospice care self-efficacy, as did those from higher-level hospitals. Medical staff might acquire relevant knowledge and skills from their own hospice care experience and then display higher hospice care self-efficacy. Medical staff from level 3 hospitals may have more access to continuous hospice education and may have better knowledge of and attitudes towards hospice care than those from lower level hospitals; this might be beneficial for their hospice care self-efficacy [48, 49]. However, studies showed Chinese health care providers in general lacked systematic and professional knowledge and skills for caring for terminal patients [50–52]. A survey investigated 141 trainees in the 2016 National Hospice and Palliative Medicine Training Program and found that only 21.3% had attended any hospice and palliative care course prior [53]. Even in the Hospice Care Department of Community Hospice Care Pilot Settings, only 50.8% medical staff had received continuous hospice care education in Shanghai [54]. Hospice care developed slowly nationwide, mainly in large cities such as Shanghai, Tianjin, and Guangzhou [55]. There remains much work to establish hospice care service with professional multi-disciplinary teams. Medical staff who assisted and worked at the COVID-19 designated hospitals in Hubei province or their own provinces were recruited from hospitals, rather than from hospice care settings. Equipped with limited knowledge and skills on hospice care, they might not be competent enough to implement hospice care. This may be one of causes of their lower level of hospice care self-efficacy.

Clinical nurses and physicians in the COVID-19 isolation wards of designated hospitals with positive coping and PAC had better hospice care self-efficacy, especially those who respected life, and had strong responsibility and professional ethics. This result was similar to that of Zheng et al. [32]. Research showed that exposure to death influenced the way health care workers perceive death [56]. Individuals with positive coping have positive thoughts and solutions (e.g. taking constructive actions and creating better living conditions and higher performance levels) [57, 58]. PAC is considered a subjective event that participates in enhancing caregiver health; PAC among caregivers is often associated with a sense of pride, self-worth, and higher self-esteem [59, 60]. In short, positive coping and PAC, as protective psychological factors, are useful for medical staff to effective deal with death work during the COVID-19 pandemic. Therefore, taking a series of measures (e.g., strengthening humanistic care, venting emotions through crying or other means rather than keeping them suppressed, improving working and rest condition, etc.) to mobilize medical staff’s positive psychological resources were critical to helping them face death or dying in fighting against the COVID-19 pandemic [61–63].

By contrast, medical staff with occupational exposure had lower hospice care self-efficacy in fighting against the COVID-19 pandemic. Health-care workers had high risk of occupational exposure to COVID-19 through intimate contact with patients with confirmed or suspected COVID-19 [64]. Medical personnel subject to blood-borne occupational exposure are easily susceptible to psychological problems and post-traumatic stress disorder, both of which are detrimental to the job performance and mental health of medical staff as well as patient outcomes [65]. Therefore, providing a safer practice environment and exploring comprehensive strategies for effective prevention and control of the occupational exposure of front-line medical staff in the fight against the COVID-19 pandemic are crucial for occupational safety and health, as well as practicing hospice care. In addition to the reasonable use of personal protective articles, it is necessary to implement more effective prevention and control measures for occupational exposure to infectious disease scientifically and in a standard fashion, as well as to intensify engineering, management, and behaviour control during prevention and control of infectious diseases [66].

The present study has some limitations. First, this is a cross-sectional study; therefore, the relationship between self-competence in death work, positive aspects of caregiving, coping strategies, and hospice care self-efficacy cannot be established. Second, based on the purpose of this study, self-reported questionnaires were used to collect the data. These methods are subject to social desirability bias [67]. Third, our study set only one question named ‘had ever given hospice care for dying or dead patients before fighting against COVID-19?’ to measure the hospice care experience of medical staff. Important as it is, the set of Chinese medical staff’s knowledge and skills in hospice care could not be included in our statistical analysis model and therefore, it might not directly reflect the inner connection between the medical staff’s skills and experience and self-efficacy in hospice care, even though the results of previous studies on this topic were cited, presumably reflected the connection in hospice care between the medical staff’s knowledge and skills and self-efficacy. Nevertheless, this study provides a foundation for future empirical research among medical staff in relation to hospice care self-efficacy and self- competence in death work and it adds to the body of knowledge on Chinese medical staff.

## Conclusion

Nurses and physicians reported a moderate level of hospice care self-efficacy during the COVID-19 pandemic. Hospice care self-efficacy was promoted by better self-competence in death work, effective coping strategies, higher level of positive aspects of caregivers, and experience of hospice care before the COVID-19 pandemic; however, it was reduced by experience of occupational exposure in fighting against the COVID-19 pandemic. Exploring the traditional Chinese philosophy of life to learn from its strengths and make up for its weaknesses and applying it to hospice care may provide a new directions for facing death or dying during the COVID-19 pandemic. Additionally, health systems could carry out continuous hospice care education to promote medical staff’s self-competence in death work by improving their hospice care knowledge and skills. Taking effective measures to mobilize positive psychological resources and providing safer practice environments to avoid occupational exposure are also essential for the improvement of the hospice care self-efficacy of nurses and physicians to effectively deal with death or dying when fighting against the COVID-19 pandemic.

## Data Availability

All datasets during and/or analysed during this study are available from the corresponding author on reasonable request.

## Abbreviations

COVID-19: Coronavirus disease 2019
CI: Confidence interval
SARS: Severe acute respiratory syndrome
MERS: Middle East respiratory syndrome
WHO: World Health Organization
NHS: National Health Commission
DMA: Disaster medical assistance
CVIs: Content validity indices
SC-DWS: Self-competence in Death Work Scale
PAC: Positive Aspects of Caregiving
SCSQ: Simplified Coping Style Questionnaire
